# Didactic program in dietetics (DPD) students’ experiences with pandemic learning and expectations for their future education: a descriptive study through a systems lens

**DOI:** 10.1186/s12909-024-05251-2

**Published:** 2024-03-06

**Authors:** Kelsey E. Mueller, Sarah G. Bellini, Emily V. Patten

**Affiliations:** Department of Nutrition, Dietetics and Food Science, Brigham Young University (BYU), 1 Campus Dr, 84604 Provo, UT USA

**Keywords:** COVID-19 education, Dietetics education, Asynchronous instruction, Synchronous instruction, Hybrid instruction, Systems approach, Higher education

## Abstract

**Background:**

The systems approach has been used to evaluate higher education and explores inputs, transformation process, and outputs of a system that is also influenced by environmental factors such as COVID-19. The COVID-19 pandemic shifted many college students to different learning modes, changing their university experience. This study evaluated dietetics students’ education experiences and characteristics in the latter period (spring 2022) of the COVID-19 pandemic using the systems approach.

**Methods:**

Researchers developed and distributed an electronic survey to all 215 US-based Didactic Program in Dietetics (DPD) directors during March to May 2022 to forward to their students. Researchers calculated descriptive statistics for variables related to inputs, transformation process, and outputs in the systems approach.

**Results:**

Respondents (*n* = 341) represented 51 DPDs in 31 states in the United States. Overall, DPD students (88.5%) were *mostly* or *very satisfied* with their choice of majoring in dietetics. Most (84.0%) planned to earn the RDN credential. Nearly half (46.9%) of DPD students were *somewhat* or *extremely concerned* about their readiness to continue their dietetics education path due to the pandemic-related learning conditions. Most students (43.6%) reported dissatisfaction with asynchronous remote instruction in laboratory courses. DPD students’ GPAs remained consistent within the range of 3.75-4.0 from Fall 2019 (43.2%) to Spring 2022 (44.5%). The most important expectations of professors moving forward were to communicate effectively (97.3%), employ cultural humility (93.8%), eliminate discrimination in the classroom (93.6%), provide lecture slides (89.7%), and be flexible and accommodating (88.7%).

**Conclusions:**

DPD students emerged from COVID-19 with new perspectives and expectations for their university learning experience. Future research should explore the perspectives of DI directors, preceptors, and employers of COVID-19 era DPD graduates.

## Background

### Dietetics education

Dietetics education prepares students to work in a variety of settings as Registered Dietitian Nutritionists (RDN) including hospitals, restaurants, universities, research, private practice counseling, sports nutrition, and more [1]. The Accreditation Council for Education in Nutrition and Dietetics (ACEND) accredits dietetics education programs [2]. While there are several pathways to becoming an RDN, each requires some combination of coursework, 1,000 h of supervised-practice, and then passing the national Registration Examination for Dietitians. Starting January 2024, a graduate degree is also required to take the national exam (3–4). In 2022, the primary pathway students used for becoming a RDN was completing a Didactic Program in Dietetics (DPD) and then a Dietetic Internship (DI) [5].

### COVID-19 pandemic and higher education

The COVID-19 pandemic shifted many college students to different learning modes, changing their university experience. During the pandemic-restriction period, instruction modes varied from synchronous (live) video instruction, asynchronous (pre-recorded) video instruction, or a hybrid blend of video and in-person instructions [6]. Less often, classes remained fully-in person with spaced-out seating [7]. The pandemic also increased use of at-home, web-camera-proctored exams [8].

College students faced negative emotional, mental, academic, and technological or other practical barriers during the pandemic period. Technological barriers included a lack of skills [9], necessity to protect students’ privacy/security [6], or internet connection on behalf of the student or professor [10]. Jowsey et al. [9] reported that nursing students believed their ability to navigate the online tools in courses affected their motivation and success, and that it was the professor’s responsibility to settle technological issues quickly. Other practical barriers for college students during the pandemic included financial stress [11], racial discrimination [11], food insecurity, job insecurity, or housing insecurity [12]. Emotional barriers included emotional instability [6], mood swings [13], loneliness, relationship issues [14], social isolation, or fear of the COVID-19 virus [11]. Mental and academic barriers included a loss of self-discipline [6], social isolation leading to anxiety and depression [14], or difficulty maintaining health behaviors [14].

Some research has shown that students became more flexible, adaptable, and resilient during the pandemic [15]. Now, college students generally prioritize meaningful relationships with peers and professors [15]; prefer certain instruction delivery modes [16]; expect technological savviness [16]; and appreciate mentor connections [17], increased flexibility [10], and accessible study materials [8].

### Theoretical framework: Systems approach

**Fig. 1:**
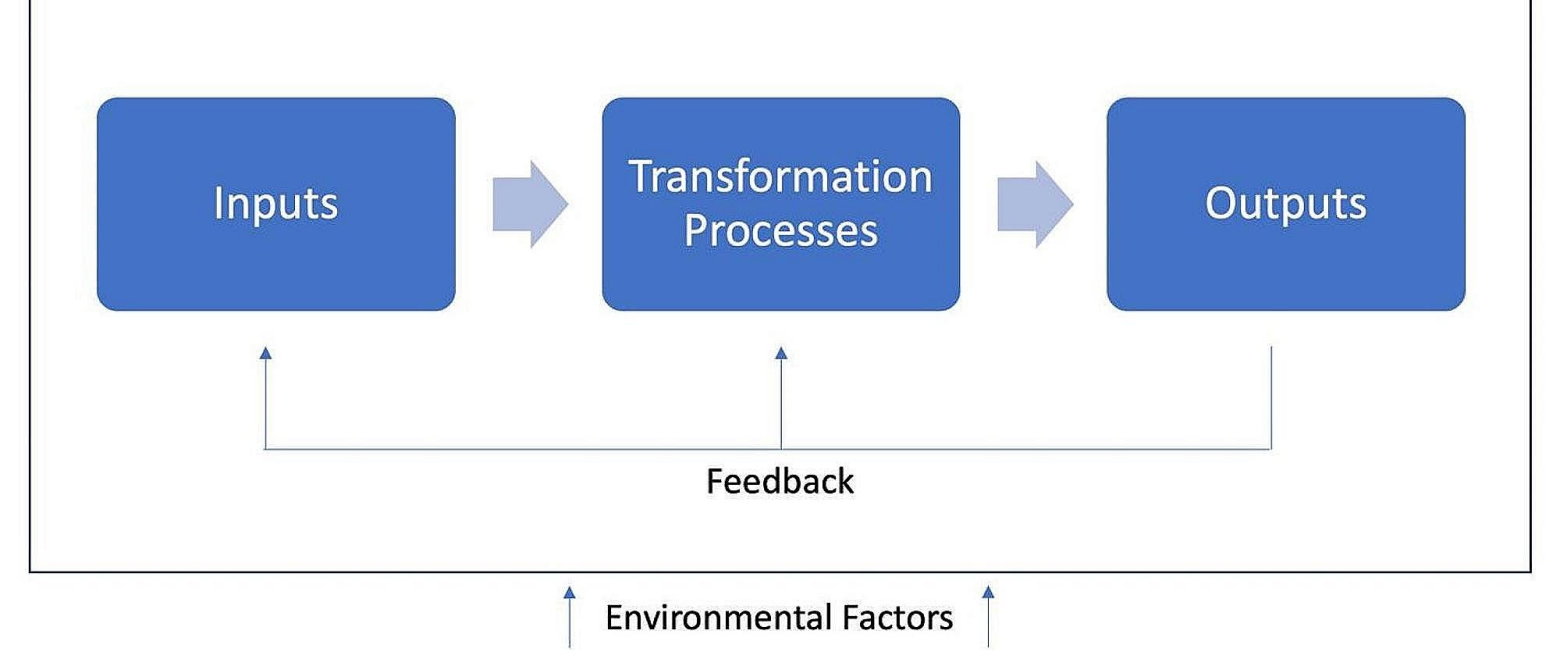
Systems approach [18–21].

A systems approach has been used to evaluate quality in higher education [18, 19]. The systems approach explores the *inputs, transformation processes, outputs*, the *feedback* process, and the influence of *environmental factors*– in this case, primarily the COVID-19 pandemic and its effects– on the system (18, 20, 21; See Fig. 1). From a technical systems perspective, higher education system *inputs* include elements like student characteristics, faculty characteristics, financial resources, facilities (including instructional equipment), programs/courses/schedules, and support services [19]. The *transformation processes* include the content, delivery, competence, attitude, assessment of needs/expectations, assessment of customer satisfaction, and management [19]. Finally, the *outputs* for a higher education system include academic achievement (e.g., success rates, skill development, competency), graduation rates, post-graduation examination pass rates, and employment achievements [19]. The purpose of this study was to evaluate the characteristics and experiences of DPD students’ in the latter period (spring 2022) of the COVID-19 pandemic using the systems approach.

## Methods

### Instrument development

This paper focuses on DPD students’ learning experiences during the pandemic and is part of a larger project exploring the overall DPD student experience with mental health, loneliness, physical wellbeing, and financial wellbeing. The sponsoring university’s Institutional Review Board granted approval for this study (#IRB2022-089).

A survey instrument previously used with this population [22, 23] was modified to include additional items exploring DPD students’ experiences with the COVID-19 pandemic. The pandemic-related items were developed based on the emerging research about college students’ experiences with pandemic learning conditions [8, 9, 12, 15, 16, 24–26]. The survey instrument included a variety of questions, including assessment of the satisfaction with dietetics as a major, expectations of professors incorporating certain practices into their teaching, satisfaction for specific learning modes for either lecture or laboratory-based courses, GPA, academic honesty, concern about their readiness to pursue dietetics, and other academic experiences and expectations. This paper will refer to “lecture-based” and “laboratory-based” classes. Lecture-based classes take place in a classroom with verbal instruction and written/typed assignments. Examples include medical nutrition therapy, introduction to nutrition or chemistry. Laboratory-based classes typically take place in rooms with hands-on instruction like microbiology lab, nutrition assessment, or student-operated restaurants/quantity food production.

Student characteristics were queried. Items included: education level, age, anticipated graduation timeline, biological sex, gender identity, ethnicity, and race. Respondents also reported which DPD they attended (presented by state and then university). One coauthor then manually coded each university as private or public.

Because the modified survey instrument included newly developed items, an expert review was conducted by university educators (*n* = 4) with expertise in survey research and dietetics education to establish content validity. Next, researchers conducted cognitive interviews with three university students to determine face validity [27]. Based on feedback from this process, researchers made minor revisions to the phrasing of seven items to improve clarity. Dietetics students (*n* = 22) from the sponsoring university pilot-tested the survey and were invited to provide feedback on the instrument. Researchers made no changes to the instrument following the pilot test; thus, the pilot respondents’ data (as originally collected) was included in the final sample.

### Data collection and analysis

In an effort to survey the entire DPD student population (*N* = 8,976 in 2022) [5], researchers sent a recruitment email with the survey instrument link to all 215 DPD Directors in the USA (sponsoring institution participated in the pilot, plus 214 DPDs) requesting them to distribute the survey to their current DPD students. Two reminder emails were sent to DPD directors. Program directors could formally opt out of receiving future emails about the study, but none did. Recruitment for this survey followed methods modeled in other studies with this population [22, 28]. Data were collected from March to May 2022. All respondents received information and gave informed consent to participate. Responding DPD students were invited to enter a drawing for a chance to receive one of seventy-five $15 Amazon gift cards. Surveys that were not at least two-thirds completed were considered incomplete and excluded from analysis. Data analysis involved calculating descriptive statistics using SPSS version 28. Means and frequencies were calculated. Chi-square analyses were conducted to identify if there were any differences based on race, ethnicity, or gender for how students rated their readiness to take further DPD coursework or to enter a dietetic internship and/or graduate program.

## Results

### Sample characteristics

Of 416 responses, ultimately, 341 were usable responses (3.8% of the total 2022 DPD population) with representation from 51 DPDs (24% of US-based DPDs). Of those, 41 were public universities and 10 private universities in 31 states. The average number of responses from each DPD was 6 but ranged from 1 to 28. Respondents primarily self-identified as female (92.0%), White (78.9%), non-Hispanic (83.9%), and non-international students (96.8%; Table 1). Of respondents, 25.5% were non-traditional learners (> 24 years) [29]. The most frequent response (46.6%) for anticipated graduation was “now through August 2022.”

**Table 1:** Didactic program in dietetics (DPD) students’ self-reported characteristics (*n* = 341)

Characteristic		n	%
*Educational Level*			
Undergraduate student		326	96.2
Graduate student		13	3.8
*Age*			
18–24 years		229	74.2
≥ 25 years		80	25.8
*Anticipated graduation timeline*			
By August 2022		158	46.6
Sep. 2022– Aug. 2023		110	32.4
Sep. 2023– Aug. 2024		48	14.2
After Aug. 2024		23	6.8
*Biological sex*			
Female		290	93.2
Male		21	6.8
*Gender identity*			
Female		286	92.0
Male		21	6.8
Other/Prefer not to disclose		4	1.3
*Hispanic or Latino origin*			
Yes		45	14.5
No		261	83.9
Prefer not to disclose		5	1.6
*Race*			
African American		10	3.2
Native Hawaiian or Pacific Islander		1	0.3
Alaskan Native or American Indian		2	0.6
Asian		14	4.5
Asian Indian		3	1.0
Multiple races		16	5.2
White		243	78.9
Other		12	3.9
Prefer not to disclose		7	2.3
*International Student*			
Yes		10	2.9
No		301	88.3
*Tested positive for COVID-19*			
Yes, once		113	34.1
Yes, more than once		38	11.5
No		171	51.7
Unsure		9	2.7
*Still experiencing COVID-19 symptoms (e.g., “long hauler”) as of spring 2022 (of those who reported testing positive for COVID-19)*			
Yes		28	18.5
No		123	81.5
*Vaccination Status*			
Partially vaccinated (1 of 2 shots)		7	2.1
Fully vaccinated (1 of 1 shots or 2 of 2 shots)		120	36.1
Fully vaccinated + booster		152	45.8
Not yet, plans to receive vaccine		11	3.3
Does NOT plan to receive vaccine		30	9.0
Prefer not to disclose		12	3.6
*Planning to become credentialled as a Registered Dietitian Nutritionist*			
Yes		284	84.0
No		20	5.9
Not sure		34	10.1
*Concern about their readiness to take further DPD coursework or enter a Dietetic Internship or Graduate program*			
Extremely concerned		41	12.3
Somewhat concerned		115	34.6
Neutral		85	25.6
Somewhat unconcerned		68	20.5
Extremely unconcerned		23	6.9
*Overall satisfaction with choosing dietetics as a major*			
Very dissatisfied		3	0.9
Mostly dissatisfied		7	2.1
Neither satisfied nor dissatisfied		29	8.6
Mostly satisfied		154	45.4
Very satisfied		146	43.1

### Educational experience

Overall, DPD students (88.5%) were *mostly* or *very satisfied* with their choice of majoring in dietetics. Most (84.0%) planned to earn the RDN credential. Nearly half (46.9%) of DPD students were *somewhat* or *extremely concerned* about their readiness to continue their dietetics education path due to the pandemic-related learning conditions. There were no significant differences based on race, ethnicity, or gender for the readiness variable.

**Fig. 2:**
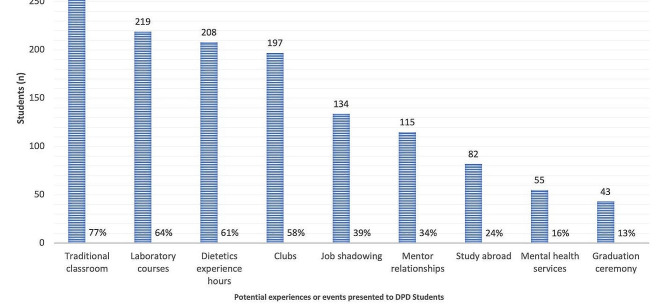
Negatively affected, canceled, or postponed experiences or events for didactic program in dietetics (DPD) students (*n* = 341)

The most reported negative disrupted experiences due to the pandemic were traditional classroom experiences (76.8%), traditional laboratory courses (64.2%), dietetics work and volunteer experiences (61.0%), and club involvement (57.8%) (Fig. 2).

DPD students’ GPAs remained consistent within the range of 3.75-4.0 from Fall 2019 (43.2%) to Spring 2022 (44.5%). Additionally, during the pandemic period, some universities offered the option for students to use a Pass/Fail mark on their transcripts rather than traditional letter grades. Half of students (48.1%) in this survey did not take the pass/fail option– only 13.0% of students reported using the pass/fail option for between one to four classes. Over a third of respondents (38.3%) reported not having that option. Few (7.1%) reported having an official academic adjustment or accommodation at their university. On a 5-point scale, respondents rated if online proctored, web-camera exams were better or worse than traditional, on-campus exams and half of respondents (50.7%) reported that online at-home exams monitored by web cameras were either *somewhat* or *much worse* for them than taking traditional, on-campus exams. Over half of respondents (56.7%) stated that they did not cheat on quizzes or exams in the past year, 11.6% cheated once per semester, and a considerable portion (19.0%) *preferred not to disclose*.

Respondents reported their satisfaction with various learning modes for lecture and laboratory-based courses they took between Winter of 2020 and Spring of 2022 (Table 2). Combining *somewhat* and *very satisfied*, most were satisfied with fully in-person laboratory-based classes (80.2%) and fully in-person lecture-based classes (80.1%). The next highest satisfaction scores for learning modes were synchronous remote instruction for lecture-based courses (69.9%) and hybrid instruction for laboratory-based courses (62.5%). Alternatively, combining *somewhat* and *very dissatisfied*, some students (43.6%) reported dissatisfaction with asynchronous remote instruction in laboratory courses. On average, 71.5% of students were satisfied at some level with the various learning modes (asynchronous, synchronous, hybrid, or fully in-person instruction) during COVID-19 for lecture-based classes, whereas fewer students (58.4%), but still many, reported satisfaction for those learning modes for laboratory-based courses.

**Table 2:** Didactic program in dietetics students’ satisfaction with learning modes used during the COVID-19 pandemic (*n* = 341)

Learning Mode	Mean ± SD	n (%)				
		Very Dissatisfied	Somewhat Dissatisfied	Neither satisfied nor Dissatisfied	Somewhat Satisfied	Very Satisfied
*Lecture-based courses* ^*a*^						
Fully in-person (*n* = 301)	4.14 ± 1.12	15 (5.0)	17 (5.6)	28 (9.3)	93 (30.9)	148 (49.2)
Synchronous remote (*n* = 316)	3.75 ± 1.14	15 (4.7)	42 (13.3)	38 (12.0)	134 (42.4)	87 (27.5)
Blended/Hybrid (*n* = 302)	3.74 ± 1.15	18 (6.0)	32 (10.6)	44 (14.6)	124 (41.1)	84 (27.8)
Asynchronous remote (*n* = 308)	3.71 ± 1.23	24 (7.8)	34 (11.0)	43 (14.0)	112 (36.4)	95 (30.8)
*Laboratory-based courses* ^*a*^						
Fully in-person (*n* = 268)	4.17 ± 1.14	14 (5.2)	15 (5.6)	24 (9.0)	73 (27.2)	142 (53.0)
Blended/Hybrid (*n* = 243)	3.59 ± 1.25	21 (8.6)	32 (13.2)	38 (15.6)	87 (35.8)	65 (26.7)
Synchronous remote (*n* = 250)	3.19 ± 1.42	39 (15.6)	54 (21.6)	38 (15.2)	59 (23.6)	60 (24.0)
Asynchronous remote (*n* = 248)	3.01 ± 1.50	55 (22.2)	53 (21.4)	33 (13.3)	49 (19.8)	58 (23.4)

^a^Five-point Scale: 1 = Very Dissatisfied to 5 = Very Satisfied; higher means indicate greater satisfaction

### Future learning expectations

Students identified potential circumstances they thought professors should accommodate with remote access to an in-person class in the future. Most commonly, students selected *health-related reasons* (45.5%), *experiencing an emergency* (45.2%), *testing positive for COVID-19* (42.2%), *planning to be absent for work, travel, etc.* (39.6%), and that access should *always be available* (38.4%). The option “*never”* was not selected by any respondents.

**Table 3:** DPD students rated the importance of various educational factors (*n* = 341)

Statement		n (%)				
	Mean ± SD	Not at all Important	Slightly Important	Moderately Important	Very Important	Extremely Important
***Professors’ practices*** ^***a***^						
Communicate effectively (*n* = 339)	4.73 ± 0.50	0 (0)	0 (0)	9 (2.7)	74 (21.8)	256 (75.5)
Eliminate discrimination in the classroom (*n* = 339)	4.72 ± 0.60	0 (0)	2 (0.6)	20 (5.9)	48 (14.2)	269 (79.4)
Exercise cultural humility in the classroom (*n* = 338)	4.62 ± 0.64	0 (0)	4 (1.2)	17 (5.0)	83 (24.6)	234 (69.2)
Provide lecture slides (*n* = 339)	4.46 ± 0.72	0 (0)	5 (1.5)	30 (8.8)	107 (31.6)	197 (58.1)
Be flexible and accommodating (*n* = 338)	4.42 ± 0.72	0 (0)	4 (1.2)	34 (10.1)	117 (34.6)	183 (54.1)
Give timely feedback on exams & assignments (*n* = 338)	4.32 ± 0.75	2 (0.6)	1 (0.3)	42 (12.4)	134 (39.6)	159 (47.0)
Connect students with dietitian mentors (*n* = 340)	4.17 ± 0.98	2 (0.6)	23 (6.8)	57 (16.8)	92 (27.1)	165 (48.7)
Provide exam study guides (*n* = 339)	4.13 ± 1.02	3 (0.9)	28 (8.3)	55 (16.2)	89 (26.3)	164 (48.4)
Connect personally with students (*n* = 339)	4.11 ± 0.90	2 (0.6)	10 (2.9)	79 (23.3)	105 (31.0)	143 (42.2)
Resolve technological issues quickly (*n* = 339)	3.90 ± 0.87	2 (0.6)	12 (3.5)	99 (29.2)	130 (38.3)	96 (28.3)
Provide access to remote class (live or recorded) (*n* = 339)	3.86 ± 1.06	6 (1.8)	32 (9.4)	85 (25.1)	95 (28.0)	121 (35.7)
***Determinants of academic success*** ^***a***^						
Computer access (*n* = 338)	4.78 ± 0.55	1 (0.3)	1 (0.3)	12 (3.6)	45 (13.3)	279 (82.5)
Internet access (*n* = 339)	4.74 ± 0.55	1 (0.3)	1 (0.3)	9 (2.7)	63 (18.6)	265 (78.2)
Feeling motivated (*n* = 339)	4.58 ± 0.63	0 (0)	2 (0.6)	20 (5.9)	96 (28.3)	221 (65.2)
Professor communication (*n* = 336)	4.52 ± 0.66	0 (0)	2 (0.6)	25 (7.4)	104 (31.0)	205 (61.0)
Stress levels under control (*n* = 338)	4.42 ± 0.81	3 (0.9)	6 (1.8)	33 (9.8)	101 (29.9)	195 (57.7)
Computer program/software access (*n* = 339)	4.40 ± 0.84	2 (0.6)	8 (2.4)	42 (12.4)	87 (25.7)	200 (59.0)
Feeling valued (*n* = 339)	4.25 ± 0.85	2 (0.6)	8 (2.4)	55 (16.2)	113 (33.3)	161 (47.5)
Human interaction (*n* = 338)	4.23 ± 0.90	3 (0.9)	10 (3.0)	57 (16.9)	103 (30.5)	165 (48.8)
Family support (*n* = 338)	4.13 ± 0.98	6 (1.8)	16 (4.7)	59 (17.5)	103 (30.5)	154 (45.6)
Access to mental health services (*n* = 339)	3.95 ± 1.03	4 (1.2)	26 (7.7)	86 (25.4)	89 (26.3)	134 (39.5)

^*a*^ Five-point Scale: 1 = Not at all important; 2 = Slightly important; 3 = Moderately important; 4 = Very important; 5 = Extremely important

Respondents rated their expectations for professors on a 5-point scale of importance (Table 3). Combining the categories *very important* and *extremely important*, students most selected *communicate effectively* (97.3%), *employ cultural humility* (93.8%), *eliminate discrimination in the classroom* (93.6%), *provide lecture slides* (89.7%), and *be flexible and accommodating* (88.7%). In another survey section (also using a 5-point scale of importance), respondents rated determinants of their academic success (Table 3). Combining *very important* and *extremely important*, students reported the highest determinants of their academic success as: internet access (96.8%), computer access (95.8%), feeling motivated (93.5%), professor communication (92.0%), and having stress levels under control (87.6%).

## Discussion

This study captured DPD students’ experiences and expectations in response to the pandemic during spring 2022. We frame our discussion using a systems approach [18, 19], specifically focusing on *inputs*, the *transformation process*, and *outputs* of the DPD education system for this sample. The environmental factors affecting the system most acutely were the COVID-19 pandemic and related mitigation efforts (e.g., social distancing, masking, university/professor policy changes, etc.).

### Inputs

An important input to this system is that each DPD was accredited by ACEND at the time of the study indicating respondents were learning from curricula consistent with established standards for dietetics education. Additionally, student characteristics were also inputs (Table 1). This sample’s racial and demographic profile is similar to the statistics reported by ACEND in 2022 for all dietetics students (this includes multiple program types, not simply DPDs); both are primarily female- and White-identifying [5]. For additional context, the most recent data indicates that the dietetics profession is 92% female and 90% White [30]. This sample is also geographically diverse within the USA with 31 states represented and 51 universities. In 2017, 4.9% of a sample of DPD students reported having an academic adjustment/accommodation [22], whereas 7.1% of DPD students in this sample reported having one. Students’ need for this academic support may have increased throughout the pandemic, or over time university students may have become more aware of how to request formal accommodations through their university.

Furthermore, respondents indicated the importance of a variety of determinants of academic success (Table 3) including computer access, internet access, computer program/software access, and professor communication. All of these are input-type variables were rated as important.

### Transformation process

In assessing the learning experiences and expectations of DPD students during this period, there were changes to typical transformation activities that potentially affected outputs in the context of this system. Here we discuss course mode and methods, professor practices, assessment, and extracurricular student development activities as transformational activities.

#### Course mode and methods

Of this sample, most respondents indicated their traditional coursework and laboratory classes were disrupted by the pandemic. More specifically, respondents reported their satisfaction with various learning modes used during that time (Table 2). For this sample, fully in-person lecture and laboratory courses had the highest satisfaction ratings. For lecture-based courses, the range of mean ratings for all learning modes was narrow suggesting the alternative options led to less variation in respondent satisfaction. For laboratory-based courses, the range of mean ratings was broader with hybrid instruction being the next highest satisfying alternative mode. Almendingen et al. [16] speculated that “Face to face training may be particularly crucial for candidates expected to have communication skills, such as nutritionists.” While all COVID waivers and flexibilities allowed by the U.S. Department of Education were rescinded October 7, 2023, educators may value how learning modes were perceived by students as they consider future course delivery modes [31]. A majority (88.8%) of students in this study felt it was important for professors to provide remote access. Students were most satisfied with synchronous followed by blended modes of remote delivery. Additionally, Edens and Kiresich [24] suggested that faculty seek further training for facilitating online courses to provide meaningful assessments, objectives, and materials in a learner-focused structure so that online education can be valuable to dietetics students. It could be that with thoughtful planning, evidence-based techniques, and adequate transition time, there may be more satisfying alternative learning modes, especially for laboratory-based courses.

#### Professor practices

Professors play a key role in course development, delivery, and management which qualify these practices as transformational activities. In this study, respondents rated communicating effectively (mean = 4.73), eliminating discrimination in the classroom (mean = 4.72), and exercising cultural humility in the classroom (mean = 4.62) as the most important professor practices. 

#### Assessment

Different ways to administer exams to assess learning came to the forefront during COVID and highlighted challenges with online exams. Half of DPD respondents (50.7%) indicated that web camera-proctored, online exams were *somewhat* or *much worse* than traditional in-person exams. Coakley and Gonzales-Pacheco [28] suggested that online test administration inaccurately evaluated and represented students’ knowledge. A quarter (24.4%) of respondents disclosed cheating *at least* once on exams and quizzes in the past year, while 19% preferred to *not disclose* their cheating record. This leads to consideration of new assessment strategies. Rogus et al. [8] stated, “Rethinking test administration by making exams open-book will reduce reliance on [various learning platform] tools with specific hardware and software requirements. This will reduce stress and allow instructors to craft questions that require application of course content to situations in the field.”

#### Extracurricular student development activities

Respondents also identified extracurricular student development activities including dietetics work and volunteer experiences, club involvement, and job shadowing as pandemic-disrupted experiences. Dietetics work/volunteer experiences and job shadowing can serve to integrate students into the field and to develop their skills and confidence. For most DPD students, completing a DI is the next step in their pathway to becoming an RDN. Importantly, many DI directors reported assessing the type and quantity of work and volunteer experience hours in admission decisions [32]. As a result of the pandemic, there may need to be allowances made for students who were not afforded these experiences. Similar literature mentioned that programs’ requirements for attaining dietetics-related work or volunteer hours were difficult for students due to the pandemic [8].

Additionally, clubs and mentorship are considered valuable to student growth. DPD students in pre-COVID-19 study reported that their universities’ student dietetics clubs were one of the leading sources for finding a mentor [23]. Other research indicates that students who serve as an officer in a club develop more leadership skills [33] and that students who participated in clubs had 24% higher job satisfaction later in life, though it did not affect occupational prestige [34].

### Outputs

From a systems perspective, these students reported several key outputs including overall GPAs, satisfaction with majoring in dietetics, post-graduation plans, and perceived readiness for continuing with next steps within dietetics education. Students in this sample maintained their GPAs in the same range throughout the pandemic experience. Interestingly, Coakley and Gonzales-Pacheco [28] shared that 43.2% of DPD and Coordinated Program in Dietetics (CPD) students reported an increase in GPAs from pre-pandemic, although 34.6% also reported earning a lower grade in a course than expected. Coakley and Gonzales-Pacheco [28] suggested that test administration may have “missed the target” in accurately assessing the students’ knowledge in virtual formats and so the increase in GPA was not a true representation of competency. Evaluation of trends on pass rates for the *Registration Examination for Dietitians* and perspectives from hiring managers of entry-level RDNs are merited to understand the long-term impact from these teaching/learning adjustments and difficulties.

Despite pandemic-related changes to the transformation process, most respondents were satisfied with selecting dietetics as a major (88.5%) and still planned to earn the RDN credential (84%). This is important considering the dietetics/nutritionist profession is growing faster than average occupations and there is a need for qualified professionals to take these new positions [35]. Notably, nearly half (46.9%) did report they were either *extremely* or *somewhat* concerned about their readiness to take further DPD coursework or enter a Dietetic Internship or Graduate Program. Dietetic internship directors/educators and preceptors may need to adjust how they train and support these students.

### Limitations

This study has limitations. While we attempted to survey the entire population, this is a convenience sample and may not be representative of the entire DPD student population in the USA. Although reflective of the ACEND’s reporting of demographic data for dietetics students [5], this may not be generalizable to all DPD students in the USA. Recruiting via a third party (DPD program directors) was also a limitation as we did not have direct communication with the potential respondents and could not remind them directly. We also only surveyed DPD students and did not include students in other types of dietetic programs; thus, this is not generalizable to all dietetics students.

## Implications and conclusions

Using a systems approach to assess the inputs, transformation process, and outputs of DPD students’ learning experience during the COVID-19 pandemic is helpful for understanding their experience, supporting them as they continue their education and credentialing, and informs future educational practices. The *inputs* in this system assessment included students’ characteristics, ACEND-accreditation, and determinants of success (e.g., device/internet access). The *transformation process* for these students was unique due to the pandemic and mitigation efforts– course modes and methods, professor practices, assessment, and extracurricular experiences were important considerations. The outputs of this system appear to be largely positive with most DPD students maintaining their GPAs throughout the pandemic, being satisfied with their choice to major in dietetics, and planning to earn the RDN credential. This research is valuable because it communicates DPD students’ satisfaction with choosing to pursue a degree in dietetics, but also their concern about readiness for their future in the field. Future research should explore the perspectives of DI directors, preceptors, and employers of COVID-19 era DPD graduates. Additionally, exploring how DPD students experience taking hybrid/blended courses post-pandemic restrictions would be helpful. If employing online learning modes with this population, we recommend thorough technological training for professors for a well-structured course, in line with ACEND’s 2022 DPD Standards [2].

## Data Availability

The datasets used and/or analyzed during the current study are available from the corresponding author on reasonable request.

## Abbreviations

DPD: Didactic Program in Dietetics
RDN: Registered Dietitian Nutritionist
ACEND: Accreditation Council for Education in Nutrition and Dietetics
DI: Dietetic Internship
